# Complete Genome Sequence of *Scardovia inopinata* JCM 12537^T^, Isolated from Human Dental Caries

**DOI:** 10.1128/genomeA.00481-15

**Published:** 2015-05-14

**Authors:** Kenshiro Oshima, Jun-ichiro Hayashi, Hidehiro Toh, Akiyo Nakano, Emi Omori, Yasue Hattori, Hidetoshi Morita, Kenya Honda, Masahira Hattori

**Affiliations:** a Graduate School of Frontier Sciences, The University of Tokyo, Kashiwa, Chiba, Japan; b CREST, Japan Science and Technology Agency, Kawaguchi, Saitama, Japan; c School of Dentistry, Aichi Gakuin University, Nagoya, Japan; d Medical Institute of Bioregulation, Kyushu University, Fukuoka, Japan; e Department of Microbiology and Immunology, Teikyo University School of Medicine, Itabashi-ku, Tokyo, Japan; f Graduate School of Environmental and Life Science, Okayama University, Okayama, Japan; g Department of Microbiology and Immunology, Keio University School of Medicine, Shinjuku-ku, Tokyo, Japan

## Abstract

*Scardovia inopinata* JCM 12537^T^ was isolated from human dental caries. Here, we report the complete genome sequence of this organism. This paper is the first report to demonstrate the fully sequenced and completely annotated genome of an *S. inopinata* strain.

## GENOME ANNOUNCEMENT

*Scardovia inopinata* JCM 12537^T^ (=DSM 10107^T^) was originally isolated as *Bifidobacterium inopinatum* from human dental caries (1) and then was renamed *S. inopinata* (2). *S. inopinata* is classified under the family *Bifidobacteriaceae*. This species is not only isolated from human dental caries and plaque (3, 4) but is also found in the human hypochlorhydric stomach (5).

We determined the complete genome sequence of *S. inopinata* JCM 12537^T^ using the whole-genome shotgun strategy using Sanger sequencing (ABI 3730xl sequencers). We constructed small-insert (2-kb) and large-insert (10-kb) genomic DNA libraries and generated 26,880 sequence reads (10.7-fold coverage) for *S. inopinata* JCM 12537^T^ from both ends of the genomic clones. The data were assembled with the Phred-Phrap-Consed program. Gap closing and resequencing of low-quality regions were conducted by Sanger sequencing to obtain the high-quality finished sequence. The overall accuracy of the finished sequence was estimated to have an error rate of <1 per 10,000 bases (Phrap score, ≥40). An initial set of predicted protein-coding genes was identified using Glimmer 3.0 (6). Genes consisting of <120 bp and those containing overlaps were eliminated. The tRNA genes were predicted by the tRNAscan-SE (7), and the rRNA genes were detected by a BLASTn search using known *Scardovia* rRNA sequences as queries.

The genome sequence of *S. inopinata* JCM 12537^T^ consists of a circular chromosome of 1,797,862 bp, with no plasmid. The genome size is smaller than those of the *Bifidobacterium* species, whose genomes range in size from 1.9 to 2.8 Mbp (8). JCM 12537^T^ contains a clustered regularly interspaced short palindromic repeat (CRISPR) (9) region (positions 468740 to 470632), and three CRISPR-associated genes (SCIP_0361 to SCIP_0363) were encoded upstream of the CRISPR region. The chromosome contains 1,445 predicted protein-coding genes. *S. inopinata* JCM 12537^T^ is related to *Parascardovia denticolens* in the phylogenetic tree of the family *Bifidobacteriaceae* (10). Next, we compared the genome of JCM 12537^T^ with that of *P. denticolens* JCM 12538^T^ (1,890,857 bp) (GenBank accession no. AP012333). Of the 1,445 protein-coding genes, 1,070 (74%) were conserved in the two strains. The genome information of this species will be useful for further studies of its physiology, taxonomy, clinical aspects, and ecology.

### Nucleotide sequence accession number. 

The sequence data for the genome have been deposited in DDBJ/GenBank/EMBL under the accession no. AP012334.
